# Burden of digestive system neoplasms in middle-aged and elderly adults: Temporal trends and geographic disparities (1990–2021)

**DOI:** 10.1371/journal.pone.0330259

**Published:** 2025-08-21

**Authors:** Yinying Chai, Tinghui Xu, Bihua Chen, Yi Wu, Yibo He, Shengliang Qiu

**Affiliations:** 1 The First School of Clinical Medicine, Zhejiang Chinese Medical University, Hangzhou, Zhejiang, China; 2 The First Affiliated Hospital of Zhejiang Chinese Medical University (Zhejiang Provincial Hospital of Chinese Medicine), Hangzhou, Zhejiang, China; Hokkaido University: Hokkaido Daigaku, JAPAN

## Abstract

**Objective:**

This study aims to comprehensively analyze the temporal trends and geographic disparities in the burden of seven digestive system neoplasms—esophageal cancer, stomach cancer, colon and rectum cancer, liver cancer, gallbladder and biliary tract cancer, pancreatic cancer, and benign and in situ intestinal neoplasms—among middle-aged and elderly populations from 1990 to 2021, and to project future trends through 2035.

**Methods:**

This study utilized data from the Global Burden of Disease (GBD) 2021 database to analyze the burden of seven digestive system neoplasms among middle-aged and elderly adults across 204 countries and territories from 1990 to 2021. Estimates of incidence, mortality, and disability-adjusted life years (DALYs) were extracted and stratified by year, sex, age group, region, and Socio-Demographic Index (SDI). Trends were evaluated using estimated annual percentage change (EAPC), and risk factors were analyzed using the GBD comparative risk assessment framework.

**Results:**

In 2021, colon and rectum cancer dominated the global burden of digestive system neoplasms. Trends from 1990 to 2021 revealed a clear split: the incidence of colon and rectum cancer (EAPC = 0.26, 95% CI 0.19 to 0.33), liver cancer (EAPC = 0.25, 95% CI 0.82 to 1.38), pancreatic cancer (EAPC = 0.55, 95% CI 0.53 to 0.57), and benign intestinal neoplasms (EAPC = 1.1, 95% CI 0.82 to 1.38) rose globally, while esophageal (EAPC = −0.92, 95% CI −1.06 to −0.79), stomach (EAPC = −1.63, 95% CI −1.71 to −1.55), and gallbladder/biliary tract cancers (EAPC = −0.28, 95% CI −0.31 to −0.25) declined. Regional hotspots for burden were East Asia and High-income Asia Pacific. Males generally faced higher burdens, except for gallbladder and biliary tract cancer. Key risk factors included smoking, alcohol consumption, dietary patterns, and metabolic factors such as high BMI and elevated fasting plasma glucose.

**Conclusions:**

The burden of digestive system neoplasms remains substantial in middle-aged and elderly adults, with significant regional differences. With the growing aging population, efforts should prioritize high-risk populations and invest in healthcare infrastructure in middle- and low-SDI regions to control established cancers. Additionally, implementing robust global prevention policies, such as expanding disease screening, raising public health awareness, and managing metabolic disorders, is essential to mitigate the rising tide of digestive system neoplasms.

## 1. Introduction

Digestive system neoplasms, encompassing esophageal cancer, stomach cancer, colorectal cancer, liver cancer, gallbladder and biliary tract cancer, pancreatic cancer, as well as benign and in situ intestinal neoplasms, are among the most pressing health concerns globally [1,2]. Digestive cancers account for approximately 26% of the global cancer incidence and 35% of cancer-related deaths each year, posing significant challenges to public health [3]. These neoplasms not only substantially reduce patients’ quality of life, shorten their life expectancy, but also impose considerable economic burdens on patients, their families, and healthcare systems [4].

Geographic disparities in the incidence, mortality, and burden of digestive system neoplasms are particularly pronounced. In 2020, the incidence of gastrointestinal cancers in East Asia accounted for 48.4% of the global total, underscoring the region’s disproportionately high burden of these diseases, with particularly high rates of stomach, liver, esophageal, and gallbladder cancers compared to other regions [5]. Contributing factors include distinct dietary patterns, lifestyle behaviors, and the higher prevalence of infectious etiologies in this region [6]. On the contrary, high-income regions such as North America have reported increasing incidences of colorectal and pancreatic cancers, which are largely associated with lifestyle factors including obesity and metabolic disorders [7,8]. At the sex level, men bear a disproportionately higher burden of digestive system neoplasms. Worldwide, distinct male-to-female incidence ratios are observed across specific subtypes of esophageal and gastric cancers, with males significantly outnumbering females in all subtypes, and the highest ratio observed for esophageal adenocarcinoma (EAC) at 6.7:1 [9]. For colorectal cancer, the incidence of colon cancer is approximately 41.2 per 100,000 person-years in men, compared to 32.4 in women, while rectal cancer incidence is 22.8 versus 12.6, respectively [10].

Epidemiological studies consistently demonstrate that the incidence and mortality of digestive system neoplasms increase with age [11,12]. For example, the incidence of colorectal cancer is concentrated in the age group of 50–69 years globally [13]. Similarly, pancreatic cancer incidence rates are relatively higher in individuals aged over 65 years, with an upward trend observed, and the average age at diagnosis ranges from 60 to 65 years [14]. And between 2000 and 2019, 81.82% of gastric cancer cases occurred in individuals aged 55 years or older [15]. Additionally, gastrointestinal cancers are often diagnosed at an advanced stages in elderly patients due to misinterpretation of symptoms [16]. Furthermore, the fact that these elderly patients often suffer from multiple chronic comorbidities, such as hypertension and diabetes, which further exacerbates the difficulties in diagnosis and treatment [17].

Although previous studies have systematically analyzed the global burden of digestive system neoplasms, research focusing specifically on middle-aged and elderly populations remains limited and has paid insufficient attention to benign and in situ intestinal neoplasms [3,18]. Additionally, elderly patients are underrepresented in clinical trials, meaning that existing treatment strategies may not fully meet their special needs. As the global population continues to age, the prevalence and burden of these diseases are expected to escalate further [19]. This study, using data from the Global Burden of Disease (GBD) 2021 database and advanced statistical methods, seeks to enhance the understanding of this field by focusing on the burden of seven digestive system neoplasms in middle-aged and elderly adults, exploring temporal trends and geographic disparities. By doing so, our study aims to provide tailored insights to inform targeted public health interventions and enhance healthcare strategies for aging populations.

## 2. Materials and methods

### 2.1 Data sources

The study utilizes the Global Burden of Disease (GBD) 2021 data retrieved from the GBD Results Tool (https://vizhub.healthdata.org/gbd-results), which encompassed 371 diseases and injuries and 88 risk factors across 204 countries and regions [20]. The dataset provided estimates of incidence, mortality, and disability-adjusted life years (DALYs) across times, sex, age group, and SDI. Our study includes all countries and regions from the GBD database for the period 1990–2021, focusing on individuals aged 55 and older, both male and female, diagnosed with digestive system neoplasms. These neoplasms include esophageal cancer, stomach cancer, colon and rectum cancer, liver cancer, gallbladder and biliary tract cancer, pancreatic cancer, and benign and in situ intestinal neoplasms. This systematic characterization of disparities in the burden of digestive neoplasms across regions and populations provides evidence-based insights to inform precision prevention strategies and healthcare prioritization.

This study quantifies the disease burden of digestive system cancers, including digestive tract cancers (esophageal cancer, stomach cancer, and colon and rectum cancer) and digestive support system cancers (liver cancer, gallbladder and biliary tract cancer, and pancreatic cancer), along with benign and in situ intestinal neoplasms, using indicators like incidence, mortality, and DALYs. Notably, DALYs are calculated as the sum of two components: years of life lost (YLLs) and years lived with disability (YLDs), with detailed definitions available in the S1 Table. Benign and in situ intestinal neoplasms are analyzed separately from malignant cancers due to their chronic, non-lethal nature and minimal impact on patients’ quality of life. Therefore, their associated DALYs and deaths are reported as 0 in the GBD study. In GBD 2021, the definitions of seven digestive system neoplasms are mapped to both the International Classification of Diseases (ICD)-10 and ICD-9 codes. The specific ICD codes corresponding to each disease are provided in the S2 Table [20].

The Socio-Demographic Index (SDI) serves as a comprehensive indicator of a country’s overall development status, incorporating multiple socio-economic parameters such as income per capita, average years of education, and fertility rates in younger females [20]. The SDI scale ranges from 0 to 1, with higher values indicating higher levels of socio-economic development. To classify countries according to their development levels, SDI values are divided into five categories based on quartiles: Low SDI (0–0.454743), Low-middle SDI (0.454743–0.607679), Middle SDI (0.607679–0.689504), High-middle SDI (0.689504–0.805129), and High SDI (0.805129–1).

### 2.2 Attributable risk factors

The comparative risk assessment framework employed in the GBD Study is designed to estimate the burden attributable to various risk factors, which are categorized into a four-level hierarchical structure. This study specifically focused on analyzing the most granular risk levels, namely levels 2 and 3, for each risk factor associated with digestive system neoplasms. In GBD 2021, the process of estimating risk factor burden involved a series of interconnected methodological steps. First, the relative risk (RR) for health outcomes was calculated based on exposure to specific risk factors. Next, exposure data were gathered and analyzed using Bayesian statistical methods to assess the distribution of risk factors. Theoretical Minimum Risk Exposure Levels (TMRELs) were then established based on the available epidemiological evidence, aiming to identify optimal exposure thresholds. Subsequently, population-attributable fractions (PAFs) were computed for each risk-outcome pair to quantify potential health improvements if exposure were reduced to the TMREL. Age-specific exposure values (SEVs) were also calculated to reflect exposure prevalence, with adjustments made for age-related risk factors. To refine these estimates, mediation factors were applied to account for indirect relationships between risk factors and outcomes. Finally, attributable burden was estimated by multiplying PAFs with the disease burden, measured in DALYs. These results were stratified by SDI quintiles and presented as counts, age-standardized rates, and rankings [21].

In our study, esophageal cancer is found to be associated with high alcohol use, smoking, chewing tobacco, and a diet low in vegetables in individuals aged 55 and older. High alcohol use is defined as alcohol consumption exceeding the TMREL, with in GBD 2021 is estimated based on region, age, sex, and year to tailor estimates to specific populations. Alcohol exposure was measured in grams of pure alcohol consumed per day. Smoking and chewing tobacco are defined as the current use of any smoked tobacco product or the use of chewing tobacco, including local products like betel quid with tobacco, in the past 30 days or according to the closest survey definition, regardless of frequency. A diet low in vegetables is defined as consuming fewer than 306–372 g/day of vegetables. Stomach cancer is linked to a diet high in sodium and smoking. High sodium intake is defined by 24-hour urinary sodium excretion levels of 1–5 g/day. Turning to colon and rectum cancer, a broader range of risk factors are identified. This included diet low in whole grains, high alcohol use, high fasting plasma glucose (FPG), smoking, diet high in red meat, diet low in calcium, diet low in fiber, diet low in milk, low physical activity, diet high in processed meat, and high body-mass index (BMI). For example, low whole grain consumption is defined as average daily consumption of fewer than 160–210 grams, while high BMI is defined as greater than 23 kg/m² for individuals aged 55 and above. Moreover, high FPG is categorized as levels above 4.88–5.30 mmol/L, and low physical activity is quantified as less than 3600–4400 MET-minutes per week. High red meat and processed meat consumption are defined as daily consumption of up to 200 grams of unprocessed red meat or any amount of processed meat. Other dietary factors, including low calcium, fiber, and milk intake, were similarly defined based on GBD 2021 thresholds (S3 Table) [21].

For liver cancer, the risk factors included smoking, high alcohol use, high FPG, drug use, and high BMI. Drug use is defined as the current use of illicit drugs, including opioids, amphetamines, and cocaine. Gallbladder and biliary tract cancer and pancreatic cancer are found to be influenced by high BMI, and pancreatic cancer is also associated with high FPG and smoking [21].

### 2.3 Statistical analysis

This study evaluated the disease burden of digestive system neoplasms among middle-aged and elderly adults, stratified by age group, sex, year, and geographical location. Temporal trends were quantified using estimated annual percentage change (EAPC) with 95% confidence intervals (CIs). Trend interpretation followed three criteria: (1) Increasing if both the EAPC and its lower CI bound exceeded 0; (2) Decreasing if both the EAPC and its upper CI bound were below 0; (3) Stable if the CI included 0. Additionally, we examined the relationship between disease-related DALY rates and SDI for each region.

To disentangle age, period, and cohort effects on epidemiological patterns, we employed the Bayesian Age-Period-Cohort (BAPC) model. This model incorporates random walk priors to smooth temporal variations while accounting for population structural changes. The model was implemented via integrated nested Laplace approximation (INLA), a computationally efficient Bayesian framework that treats parameters as random variables with specified prior distributions [22]. Compared to other methods, the BAPC model exhibits superior robustness in handling sparse data and projecting age-standardized trends.

Data analysis and visualization were conducted using R software (version 4.4.1), with the following packages employed for data manipulation, statistical analysis, and visualization: “tidyverse”, “dplyr”, “ggplot2”, “ggmap”, “sf”, “BAPC”, and “INLA”.

## 3. Results

### 3.1 Digestive system neoplasms in middle-aged and elderly adults: Global trends

In 2021, the global incidence was estimated at approximately 488 thousand people (95% uncertainty interval [UI] 429–548) for esophageal cancer, 1,020 thousand people (95% UI 868–1,161) for stomach cancer, and 1.83 million people (95%UI 1.66 to 1.96) for colon and rectum cancer (Table 1). Colon and rectum cancer had the highest incidence rate at 123.13 per 100,000 (95%UI 111.87 to 132.19). From 1990 to 2021, colon and rectum cancer (EAPC = 0.26, 95% CI 0.19 to 0.33) showed an increasing trend. Conversely, both esophageal cancer (EAPC = −0.92, 95% CI-1.06 to −0.79), and stomach cancer (EAPC = −1.63, 95% CI −1.71 to −1.55) experienced a decline in incidence rates (Table 1). In the same year, the global incidence was estimated to be around 401 thousand people (95%UI 365–444) for liver cancer, 192 thousand people (95%UI 163–217) for gallbladder and biliary tract cancer, and 448 thousand people (95%UI 405–483) for pancreatic cancer (Table 1). From 1990 to 2021, gallbladder and biliary tract cancer (EAPC = −0.28, 95% CI-0.31 to −0.25) incidence showed a decreasing trend, while liver cancer (EAPC = 0.25, 95% CI 0.82 to 1.38) and pancreatic cancer (EAPC = 0.55, 95% CI 0.53 to 0.57) displayed increasing trends (Table 1).

**Table 1:** Global incidence, mortality, and DALYs of digestive system neoplasms in middle-aged and elderly adults in 1990/2021 and their temporal trends.

	1990		2021		1990–2021 EAPC (95%CI)
	Counts (95%UI)	Rate (95%UI)	Counts (95%UI)	Rate (95%UI)	
**Incidence**					
Esophageal cancer	276819 (246623-303205)	41.23 (36.73-45.16)	487973 (429298-548485)	32.84 (28.89-36.91)	−0.92 (−1.06 to −0.79)
Stomach cancer	748262 (685876-820446)	111.44 (102.15-122.19)	1019630 (868077-1160558)	68.62 (58.42-78.1)	−1.63 (−1.71 to −1.55)
Colon and rectum cancer	744710 (700000-772875)	110.91 (104.26-115.11)	1829732 (1662320-1964373)	123.13 (111.87-132.19)	0.26 (0.19 to 0.33)
Liver cancer	161130 (148984-176801)	24 (22.19-26.33)	401168 (364558-443755)	27 (24.53-29.86)	0.25 (0.12 to 0.38)
Gallbladder and biliary tract cancer	92729 (83666-100988)	13.81 (12.46-15.04)	192028 (163007-216508)	12.92 (10.97-14.57)	−0.28 (−0.31 to −0.25)
Pancreatic cancer	173675 (164223-181689)	25.87 (24.46-27.06)	448383 (404923-482978)	30.17 (27.25-32.5)	0.55 (0.53 to 0.57)
Benign and in situ intestinal neoplasms	336899 (236325-484143)	50.18 (35.2-72.11)	1038322 (744431.77-1431781)	69.87 (50.1-96.35)	1.1 (0.82 to 1.38)
**Mortality**					
Esophageal cancer	286544 (255217-313422)	42.68 (38.01-46.68)	467907 (412193-525086)	31.49 (27.74-35.34)	−1.2 (−1.35 to −1.05)
Stomach cancer	675865 (618067-748598)	100.66 (92.05-111.49)	816991 (698480-925626)	54.98 (47-62.29)	−2.05 (−2.15 to −1.95)
Colon and rectum cancer	475987 (445350-497536)	70.89 (66.33-74.1)	908497 (820975-975994)	61.14 (55.25-65.68)	−0.57 (−0.62 to −0.51)
Liver cancer	165023 (152295-181899)	24.58 (22.68-27.09)	383399 (349408-423450)	25.8 (23.51-28.5)	0.06 (−0.07 to 0.19)
Gallbladder and biliary tract cancer	86386 (77436-95544)	12.87 (11.53-14.23)	155077 (129703-174512)	10.44 (8.73-11.74)	−0.77 (−0.81 to −0.73)
Pancreatic cancer	180634.91 (170685-189026)	26.9 (25.42-28.15)	453010 (409091-487561)	30.49 (27.53-32.81)	0.44 (0.42 to 0.46)
Benign and in situ intestinal neoplasms	NA	NA	NA	NA	NA
**DALYs**					
Esophageal cancer	6735509 (5994877-7409223)	1003.17 (892.86-1103.51)	9991156 (8805232-11251749)	672.36 (592.55-757.19)	−1.5 (−1.63 to −1.37)
Stomach cancer	15165828 (13733880-16789771)	2258.75 (2045.48-2500.62)	16668809 (14297014-19121976)	1121.73 (962.12-1286.82)	−2.37 (−2.46 to −2.29)
Colon and rectum cancer	9999397 (9452012-10456034)	1489.28 (1407.75-1557.29)	18176256 (16712440-19425789)	1223.18 (1124.67-1307.27)	−0.74 (−0.79 to −0.69)
Liver cancer	3908185 (3603136-4308069)	582.07 (536.64-641.63)	8260582 (7548135-9176565)	555.9 (507.95-617.54)	−0.26 (−0.37 to −0.15)
Gallbladder and biliary tract cancer	1787213 (1617188-1980946)	266.18 (240.86-295.04)	2999189 (2489403-3445819)	201.83 (167.53-231.89)	−1.01 (−1.05 to −0.96)
Pancreatic cancer	3848534 (3667147-4026315)	573.19 (546.17-599.67)	9042586 (8338981-9697828)	608.52 (561.17-652.62)	0.22 (0.2 to 0.24)
Benign and in situ intestinal neoplasms	0 (0−0)	0 (0−0)	0 (0−0)	0 (0−0)	NA

DALYs, disability-adjusted life years; EAPC, estimated annual percentage change; UI, uncertainty Interval; CI, confidence interval.

In terms of mortality in 2021, esophageal cancer accounted for roughly 468 thousand people (95%UI 412–525), stomach cancer for 817 thousand people (95%UI 698–926), and colon and rectum cancer for 908 thousand people (95%UI 821–976) (Table 1). Notably, all three digestive tract cancers demonstrated declining mortality trends from 1990 to 2021, with stomach cancer (EAPC = −2.05, 95% CI −2.15 to −1.95) showing the most pronounced decrease (Table 1). Pancreatic cancer had the highest mortality rate in 2021 at 30.49 per 100,000 (95%UI 27.53 to 32.81) compared to the other two types of digestive support system cancers (Table 1). Over the course of 32 years, pancreatic cancer (EAPC = 0.44, 95% CI 0.42 to 0.46) showed upward mortality trends, whereas liver cancer (EAPC = 0.06, 95% CI −0.07 to 0.19) mortality remained relatively stable, and a decreasing trend was observed for gallbladder and biliary tract cancer (EAPC = −0.77, 95% CI −0.81 to −0.73)(Table 1).

In 2021, colon and rectum cancer accounted for the highest number of DALYs among digestive system neoplasms globally, at about 18.18 million people (95%UI 16.71 to 19.43) (Table 1). From 1990 to 2021, the DALY rate for esophageal cancer (EAPC = −1.5, 95% CI −1.63 to −1.37) showed a declining trend, as did stomach cancer (EAPC = −2.37, 95% CI −2.46 to −2.29) and colon and rectum cancer (EAPC = −0.74, 95% CI −0.79 to −0.69)(Table 1). Among digestive support system cancers, pancreatic cancer had the highest DALY rate in 2021, at 608.52 (561.17 to 652.62) (Table 1). Furthermore, from 1990 to 2021, pancreatic cancer (EAPC = 0.22, 95% CI 0.2 to 0.24) experienced an increase in DALYs, while liver cancer (EAPC = −0.26, 95% CI −0.37 to −0.15) and gallbladder and biliary tract cancer (EAPC = −1.01, 95% CI −1.05 to −0.96) showed decreasing trends (Table 1).

### 3.2 Digestive system neoplasms in middle-aged and elderly adults: Regional level

When analyzed by geographic region in 2021, East Asia experienced the greatest burden in terms of incidence, mortality, and DALYs across most digestive tract cancers. The incidence rate of esophageal cancer was highest in East Asia (72.13 per 100,000, 95%UI 58.14 to 88.08) and lowest in Northern Africa (4.28 per 100,000, 95%UI 3.57 to 5.05), representing an 18-fold difference. The greatest incidence rates for stomach cancer (162.52 per 100,000, 95%UI 139.61 to 176.03) and colon and rectum cancer (269.48 per 100,000, 95%UI 230.76 to 291.57) were observed in the High-income Asia Pacific (S4 Table). The mortality rates for esophageal cancer (68.69 per 100,000, 95%UI 55.37 to 83.30), stomach cancer (105.48 per 100,000, 95%UI 85.5 to 129.07), and colon and rectum cancer (131.12 per 100,000, 95%UI 120.28 to 140.73) peaked in East Asia, Andean Latin America, and Central Europe, respectively (S5 Table). As for DALYs, East Asia reported the highest rates for esophageal cancer (1448.42 per 100,000, 95% UI 1159.59 to 1776.84) and stomach cancer (2102.28 per 100,000, 95% UI 1625.85 to 2617.69). The corresponding lowest rates were found in Northern Africa (103.15 per 100,000, 95% UI 84.99 to 122.23) and North America (289.23 per 100,000, 95% UI 269.07 to 303.79), while the highest rates in East Asia were nearly 14 and 7 times higher. Meanwhile, Central Europe had the highest DALY rate for colon and rectum cancer (2546.30 per 100,000, 95% UI 2347.63 to 2735.28) (S1 Table).

**Fig 1:**
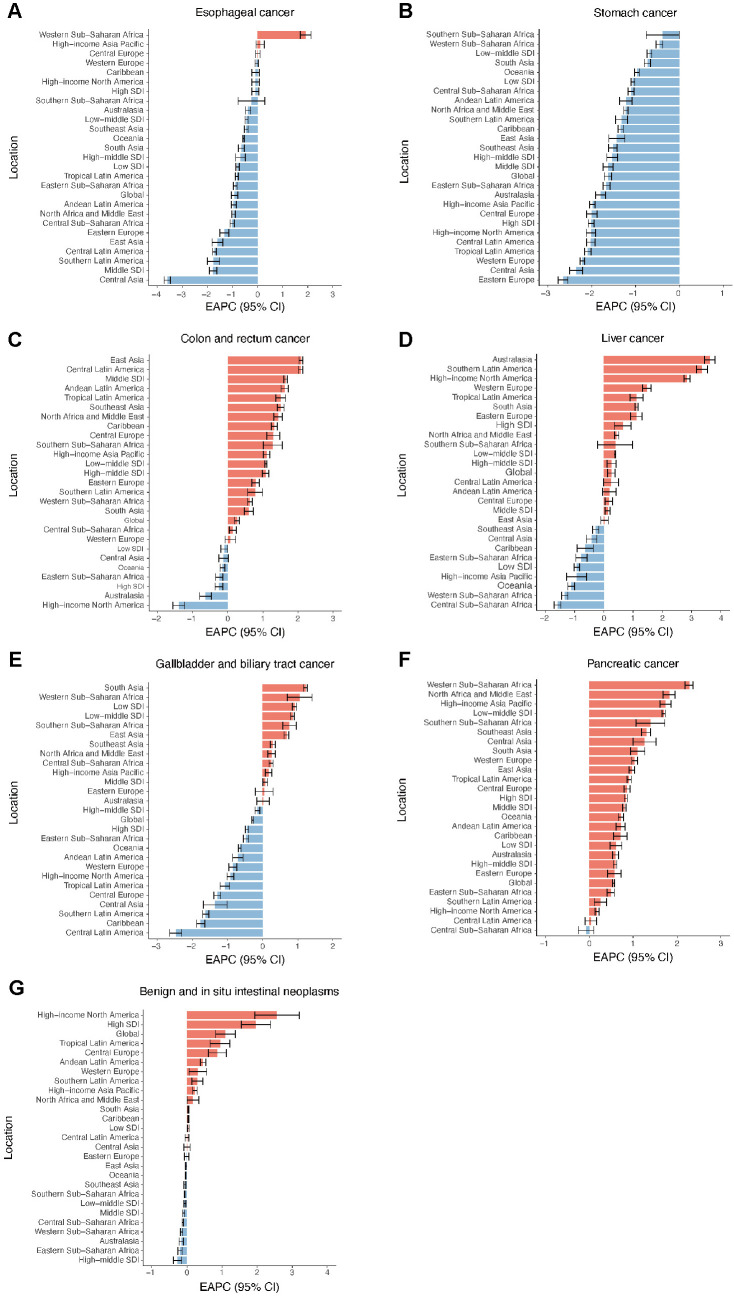
EAPC of incidence rates for digestive system neoplasms in global and regional populations of middle-aged and elderly adults (1990–2021). (A) Esophageal cancer. (B) Stomach cancer. (C) Colon and rectum cancer. (D) Liver cancer. (E) Gallbladder and biliary tract cancer. (F) Pancreatic cancer. (G) Benign and in situ intestinal neoplasms.

From 1990 to 2021, the burden of esophageal cancer decreased in most GBD regions, especially in Central Asia, while Western Sub-Saharan Africa showed significant rises for incidence, mortality, and DALY rates (Figs 1[incidence], S1[death] and S2[DALY]). Notably, stomach cancer demonstrated decreasing trends in the rates of incidence, mortality and DALY across all GBD regions, particularly pronounced in Eastern Europe (Figs 1, S1 and S2). Colon and rectum cancer saw an increase in incidence and death rates in most regions, especially in Central Latin America. However, the DALY rates have decreased in more than half of the regions, with the largest reductions observed in Australasia (Figs 1, S1 and S2).

In contrast to digestive tract cancers, the High-income Asia Pacific exhibited the highest incidence, mortality, and DALY rates for most digestive support system cancers, with the exception of pancreatic cancer, whose DALY rate peaked in Central Europe. Detailed estimates are provided in S1–S3 Tables. By comparison, the lowest incidence (8.43 per 100,000, 95% UI 7.31 to 9.57), mortality (9.19 per 100,000, 95% UI 8.03 to 10.42), and DALY rate (194.85 per 100,000, 95% UI 168.95 to 223.35) for liver cancer were observed in the Caribbean. The lowest incidence (0.24 per 100,000, 95% UI 0.16 to 0.29) and mortality (0.26 per 100,000, 95% UI 0.18 to 0.32) rates of gallbladder and biliary tract cancer were both observed in Western Africa, representing over 210-fold and nearly 150-fold differences compared to the corresponding rates in the High-income Asia Pacific. Remarkably, the lowest incidence (6.76 per 100,000, 95% UI 6.02 to 7.47), mortality (7.29 per 100,000, 95% UI 6.51 to 8.04), and DALY (164.09 per 100,000, 95% UI 146.52 to 181.14) rates of pancreatic cancer in 2021 were all found in South Asia (S1–S3 Tables).

From 1990 to 2021, there was a remarkable rise in rates of incidence and mortality for liver cancer across numerous regions, particularly pronounced in Southern Latin America, and Australasia. Conversely, a decline was observed for DALY rate in over half of regions, with the most significant reductions occurring in High income Asia Pacific (Figs 1, S1 and S2). The incidence, mortality, and DALY rates for gallbladder and biliary tract cancer have declined over time in most regions, particularly in Central Latin America (Figs 1, S1 and S2). Pancreatic cancer demonstrated increasing trends at all rates across the vast majority of regions, with Western Sub to Saharan Africa experiencing the most significant increases in pancreatic cancer burden (Figs 1, S1 and S2).

### 3.3 Digestive system neoplasms in middle-aged and elderly adults: National trends

**Fig 2:**
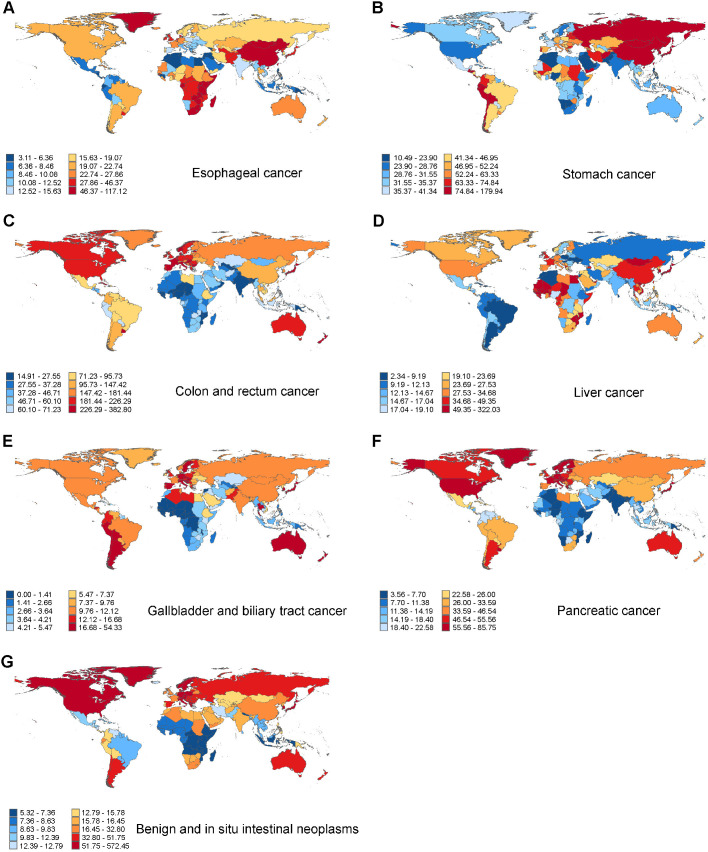
Incidence rates of digestive system neoplasms in middle-aged and elderly adults per 100,000 population for both sexes in 204 countries, in 2021. (A) Esophageal cancer. (B) Stomach cancer. (C) Colon and rectum cancer. (D) Liver cancer. (E) Gallbladder and biliary tract cancer. (F) Pancreatic cancer. (G) Benign and in situ intestinal neoplasms.

In 2021, digestive tract cancers exhibited substantial geographic disparities across countries. For esophageal cancer, Malawi recorded the highest incidence (117.11 per 100,000, 95% UI 93.27 to 145.03), mortality (125.11 per 100,000, 95% UI 99.78 to 155.33), and DALY rate (3018.62 per 100,000, 95% UI 2387.41 to 3771.59), while Algeria reported the lowest (Figs 2[incidence], S3[death] and S4[DALY]). In stomach cancer, the highest incidence was observed in Japan (179.93 per 100,000, 95% UI 153.67 to 194.68), whereas Mongolia showed the highest mortality (154.55 per 100,000, 95% UI 120.76 to 189.65) and DALY (3664.72 per 100,000, 95% UI 2886.72 to 4535.68) rates. Conversely, Nigeria had the lowest incidence, and Kuwait reported the lowest mortality and DALY rates (Figs 2, S3 and S4). For colon and rectum cancer, the Netherlands had the highest incidence (382.80 per 100,000, 95% UI 337.82 to 424.41), Uruguay had the highest mortality rate (169.05 per 100,000, 95% UI 147.97 to 191.13), and Hungary had the highest DALY rate (3051.51 per 100,000, 95% UI 2584.24 to 3644.17). The lowest incidence and DALY rates were seen in Gambia, while Papua New Guinea had the lowest mortality (Figs 2, S3 and S4).

Mongolia ranked highest for liver cancer in all three measures: incidence (322.02 per 100,000, 95% UI 242.07 to 410.10), mortality (347.24 per 100,000, 95% UI 261.30 to 442.38), and DALYs (8345.79 per 100,000, 95% UI 6281.83 to 10643.69), whereas Morocco had the lowest burden (Figs 2, S3 and S4). In gallbladder and biliary tract cancer, Japan reported the highest incidence (54.33 per 100,000, 95% UI 44.02 to 60.38) and mortality (41.73 per 100,000, 95% UI 33.88 to 46.32), while Chile had the highest DALY rate (805.37 per 100,000, 95% UI 736.10 to 827.70); Gambia recorded the lowest values for all three metrics (Figs 2, S3 and S4). For pancreatic cancer, the highest incidence, mortality, and DALY rates were observed in Japan (85.74 per 100,000, 95% UI 71.50 to 93.80), Monaco (84.82 per 100,000, 95% UI 55.82 to 123.73), and Greenland (1617.54 per 100,000, 95% UI 1303.61 to 1975.32), while Mozambique showed the lowest rates across all indicators (Figs 2, S3 and S4).

### 3.4 Digestive system neoplasms in middle-aged and elderly adults: Age and sex patterns

**Fig 3:**
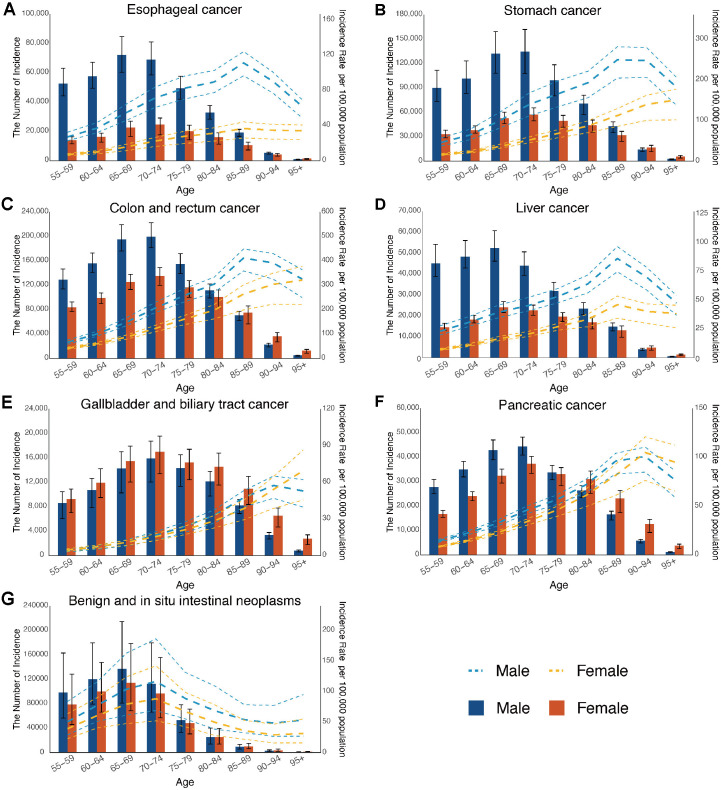
Global counts and incidence rates of digestive system neoplasms in middle-aged and elderly adults by age and sex, 2021. (A) Esophageal cancer. (B) Stomach cancer. (C) Colon and rectum cancer. (D) Liver cancer. (E) Gallbladder and biliary tract cancer. (F) Pancreatic cancer. (G) Benign and in situ intestinal neoplasms.

In 2021, esophageal cancer showed a significant sex gap in the cases of incidence, deaths, and DALYs started from the 55–79 years. Globally, males had significantly higher incidence, mortality, and DALY rates than females. The highest incidence, mortality, and DALYs of stomach cancer were concentrated in individuals aged between 65–74 years, with males exhibiting higher rates across all age groups above 55. In colon and rectum cancer, females had higher numbers of cases, deaths, and DALYs than males over the age of 85. For males, the incidence and deaths in males were predominantly observed in the 70–74 age group, while the DALYs were concentrated in the 65–69 age group. The highest rates of incidence, mortality and DALY were recorded in the 85–89 and 90–94 age groups for males. Additionally, in both stomach cancer and colon and rectum cancer, these rates increased progressively with age in females (Figs 3[incidence], S5[death] and S6[DALY]).

In 2021, liver cancer incidence, deaths, and DALYs are most concentrated in the 65–69 age group, although the DALYs for males declines with age. Males’ rates of incidence, mortality, and DALYs peaked in the 85–89, 90–94, and 65–69 age groups, respectively, while females’ corresponding maxima were in the 85–89, ≥ 95, and 90–94 age groups. Females constituted the primary demographic affected by gallbladder and biliary tract cancer with higher incidence, deaths and DALYs compared to males. While the incidence, mortality, and DALY rates for females increased with advancing age, males had the highest rates in the 90–94 age group. The incidence and mortality of pancreatic cancer for both males and females were concentrated in the 70–74 age group, whereas the DALY numbers were primarily in the 65–69 age group. The peak incidence of pancreatic cancer occurred in the 90–94 age group. Males had the highest mortality and DALY rates in the 90–94 and 85–89 age groups, while females experienced a steady increase in these rates with age (Figs 3, S5 and S6).

### 3.5 Burden of digestive system neoplasms in middle-aged and elderly adults by SDI

**Fig 4:**
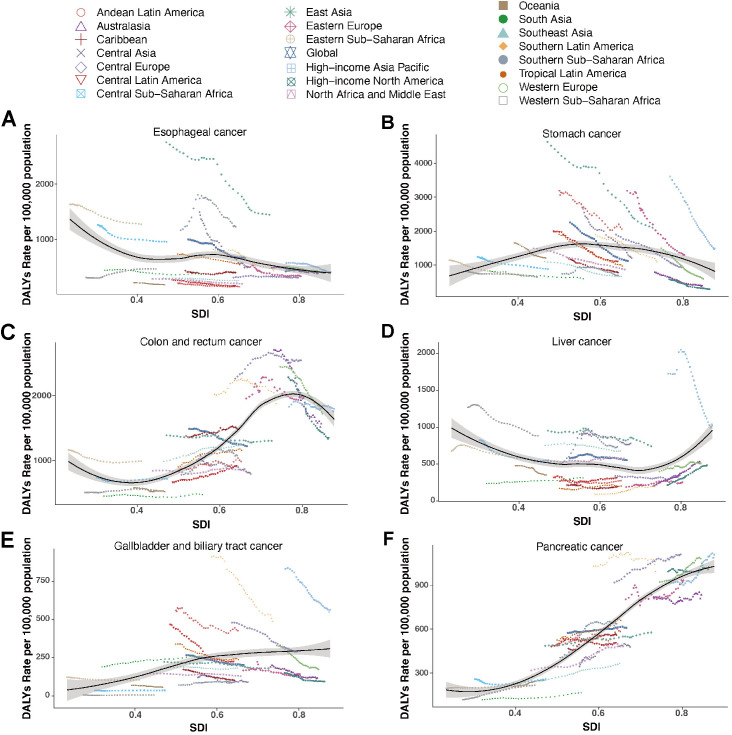
DALY rates for digestive system neoplasms in middle-aged and elderly adults for 21 global burden of disease regions from 1990 to 2021 by SDI. (A) Esophageal cancer. (B) Stomach cancer. (C) Colon and rectum cancer. (D) Liver cancer. (E) Gallbladder and biliary tract cancer. (F) Pancreatic cancer.

In 2021, DALY rates for Digestive System Neoplasms exhibited distinct patterns across the SDI spectrum. The high and high-middle SDI quintile levels exhibited higher DALY rates of esophageal cancer (878.07 per 100,000, 95%UI 725.17 to 1065.11), stomach cancer (1525.78 per 100,000, 95%UI 1267.50 to 1792.53) and colon and rectum cancer (1637.44 per 100,000, 95%UI 1488.96 to 1735.87) (S1 Table). For esophageal cancer, DALY rates generally declined as SDI increased, with the sharpest drop observed in East Asia. Stomach cancer displayed an initial rise in DALY rates with increasing SDI, followed by a decline in higher-SDI regions. In particular, East Asia, Andean Latin America, Eastern Europe and High-income Asia Pacific had stomach cancer burdens exceeding SDI-based expectations. For colon and rectum cancer, the DALY rates initially decreased with rising SDI, then increased and peaked at a middle-to-high SDI level, followed by a renewed decline as SDI continued to rise (Fig 4). From 1990 to 2021, DALY rates for esophageal cancer declined most in middle SDI, while the largest reductions for stomach cancer and colon and rectum cancer were observed in high SDI (S2 Fig).

Liver cancer (598.67 per 100,000, 95%UI 553.10 to 633.69), gallbladder and biliary tract cancer (238.21 per 100,000, 95%UI 208.79 to 260.77), and pancreatic cancer (1031.69 per 100,000, 95%UI 945.24 to 1094.24) showed higher rates in the high SDI quintile levels (S4 Table). In the case of liver cancer, a U-shaped trend was observed, where both high- and low-SDI regions experienced elevated DALY rates, with High-income Asia Pacific bearing the highest burden. For gallbladder and biliary tract cancer, DALY rates increased with SDI, although regions such as High-income Asia Pacific and Southern Latin America showed markedly declining trends. Pancreatic cancer demonstrated a steady rise in DALY rates with increasing SDI. Central Europe and Southern Latin America showing higher values than SDI-based expectations, whereas South Asia consistently remained below expectations (Fig 4). From 1990 to 2021, DALY rates for liver cancer declined most notably in low SDI, while gallbladder and biliary tract cancer showed the greatest reductions in high SDI. In contrast, pancreatic cancer exhibited a more pronounced upward trend in low-middle SDI (S2 Fig).

The results of the global, regional, and national trends, as well as the age and sex patterns of benign and in situ intestinal neoplasms, are detailed in S1 File.

### 3.6 Risk factors of digestive system neoplasms in middle-aged and elderly adults

Previous research has explored the association between risk factors and digestive system neoplasms, with consistent and region-specific findings. A pooled analysis of data from 10 case–control studies on EAC revealed that smoking doubled the risk of this malignancy [23]. Consistent with this, our results identified smoking as the leading global risk factor for esophageal cancer, with particularly high proportional contributions in East Asia and High-income North America. Notably, in South Asia, chewing tobacco emerged as a significant risk factor for esophageal cancer. For gastric cancer, a 2020 meta-analysis encompassing 232 studies found that current smoking increased the odds of developing gastric cancer by 61% [24]. Our study further confirmed that smoking and a high-sodium diet were the most critical global risk factors for gastric cancer, with more pronounced impacts observed in East Asia and High-income North America. In the case of colon and rectum cancer, dietary factors were particularly prominent. Low intake of whole grains, milk, and calcium, coupled with high red meat consumption, were major global contributors to the disease burden. Additionally, BMI and FPG exerted substantial impacts, especially in High-income North America and Central Latin America.

**Fig 5:**
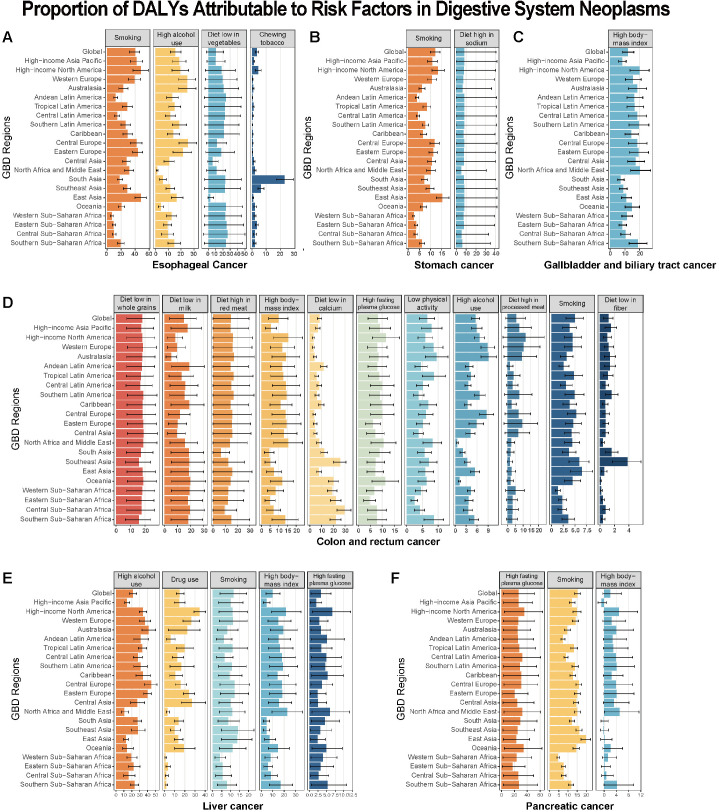
Percentage of digestive system neoplasms in middle-aged and elderly adults DALYs attributable to risk factors. (A) Esophageal cancer. (B) Stomach cancer. (C) Gallbladder and biliary tract cancer. (D) Colon and rectum cancer. (E) Liver cancer. (F) Pancreatic cancer.

Liver cancer showed a strong association with alcohol use, with the highest proportional contribution observed in Central Europe. For gallbladder and biliary tract cancer, high BMI was the most influential factor across regions, with North Africa and the Middle East experiencing the highest burdens. A meta-analysis of 14 cohort studies confirmed that overweight individuals had a 1.10-fold higher relative risk of gallbladder cancer (95% CI: 1.02–1.18), and obesity was associated with a 1.69-fold increased risk (95% CI: 1.54–1.86), showing a dose-response relationship [25]. For pancreatic cancer, high fasting plasma glucose, smoking, and high BMI emerged as three key risk factors worldwide. Interestingly, high BMI appeared to act as a protective factor in some regions. Specifically, in South Asia, Southeast Asia, and High-income Asia Pacific, the attributable DALYs were −9.20 × 10 ⁻ ⁴(95% UI: −9.88 × 10 ⁻ ³ to 1.31 × 10 ⁻ ²), −1.51 × 10 ⁻ ³ (95% UI: −1.14 × 10 ⁻ ² to 1.45 × 10 ⁻ ²), and −8.83 × 10 ⁻ ³ (95% UI: −2.02 × 10 ⁻ ² to 7.38 × 10 ⁻ ³), respectively. However, since the uncertainty intervals crossed zero, the significance of this protective effect remains questionable (Fig 5).

### 3.7 Predictions of digestive system neoplasms in middle-aged and elderly adults from 2020 to 2035

**Fig 6:**
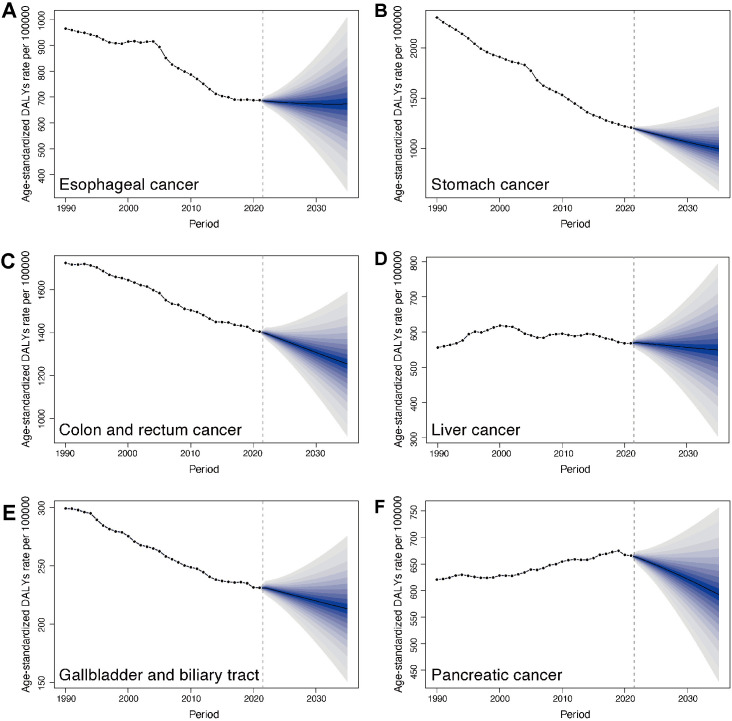
Temporal trends of age-standardized DALY rates for digestive system neoplasms in middle-aged and elderly adults (1990–2035). (A) Esophageal cancer. (B) Stomach cancer. (C) Colon and rectum cancer. (D) Liver cancer. (E) Gallbladder and biliary tract cancer. (F) Pancreatic cancer.

We projected global age-standardized incidence rate (ASIR), age-standardized death rate (ASDR) and age-standardized DALY rate for digestive system neoplasms in middle-aged and elderly adults from 2020 to 2035 by using the BAPC model. From 2020 to 2035, ASIR of most digestive system neoplasms are projected to decline (S7 Fig). However, esophageal cancer stands out with a continued upward trend in ASIR during the prediction period, in contrast to the other neoplasms. Benign and in situ intestinal neoplasms demonstrated the most pronounced decrease in ASIR, reversing the rapid growth seen in previous decades. ASDR for all six major digestive system cancers demonstrated consistent declines (S8 Fig). Among these, stomach cancer and colon and rectum cancer exhibited the most substantial mortality decreases. Similarly, the age-standardized DALY rates showed declining trends across all digestive system neoplasms, while esophageal and liver cancers displayed slower declines in disease burden (Fig 6).

## 4. Discussion

This study comprehensively analyzes the global burden and temporal trends of seven major digestive system neoplasms among middle-aged and elderly populations from 1990 to 2021. The findings highlight distinct patterns in disease distribution, risk factor impacts, and evolving trends, with critical implications for public health strategies.

In 2021, the majority of cases, deaths, and DALYs associated with these cancers were concentrated in individuals aged 65–74, with incidence rates generally increasing with age. This age-related trend may be partially linked to immunosenescence, long-term cumulative exposure to risk factors, and the accumulation of chronic inflammation [26,27].

Males consistently exhibited higher incidence, mortality, and DALY burdens for most digestive system neoplasms, particularly esophageal, stomach, and liver cancers. This disparity was likely associated with the higher prevalence of high-risk behaviors among males, such as tobacco use, alcohol consumption, unhealthy dietary patterns, and insufficient physical activity [28]. Moreover, higher concentrations of sex hormone-binding globulin (SHBG) and testosterone have been linked to increased gastric and liver cancer risks in men [29]. In contrast, females exhibited higher rates of gallbladder and biliary tract cancers, which may partly be attributed to the higher occurrence of gallstones, a recognized risk factor for gallbladder cancer [30,31]. Furthermore, some studies suggest that reproductive and menstrual factors may be linked to gallbladder cancer, highlighting the potential role of female hormones in its etiology. However, the findings remain inconsistent, necessitating further research to clarify the specific mechanisms underlying their role [32,33]. These sex-specific disparities in digestive system cancers necessitate sex-responsive prevention and intervention strategies. For males, public health policies should prioritize primary prevention, such as intensifying tobacco and alcohol control measures and encouraging regular physical activity. For females, more proactive screening and monitoring of gallbladder and biliary tract diseases are essential, particularly for individuals with gallstones or relevant reproductive histories. Such tailored approaches are crucial to improving the precision and effectiveness of efforts to control digestive system neoplasms.

Tobacco use, alcohol consumption, dietary patterns, and elevated FPG are major global risk factors for digestive system neoplasms, with their impact and distribution varying significantly across different SDI levels. Smoking, a leading risk factor for esophageal cancer, exerts carcinogenic effects through mechanisms such as inducing genetic mutations in esophageal cells, promoting chronic inflammation-mediated epithelial remodeling, and hurting the immune system [34,35]. In East Asia, persistently high male smoking rates likely sustain the region’s elevated esophageal cancer burden despite global declines [36]. In addition, the use of betel nut-tobacco mixtures remained prevalent in certain South Asian countries, resulting in persistent direct contact between carcinogens the esophageal mucosa [37]. These compounds may synergistically increase the risk of esophageal cancer through mechanisms such as hypermethylation of tumor suppressor gene promoters like p16 and genetic mutations in genes such as p53 [38]. Additionally, our results indicate that lifestyle factors, particularly a high-sodium diet, contribute significantly to the burden of stomach cancer in middle-aged and elderly populations, especially in East Asian countries. Reducing the intake of high-salt foods has been proposed as one strategy to address the stomach cancer issue in high-risk Asian countries [39].

Dietary factors play a central role in colorectal cancer (CRC), particularly a low intake of whole grains and a high intake of red and processed meats. This has been confirmed by the National Institutes of Health and American Association of Retired Persons (NIH-AARP) Diet and Health Study, which found that whole grain consumption, but not dietary fiber from other sources, is inversely associated with CRC risk [40]. Additionally, a high intake of red and processed meats has been shown to increase CRC risk through mechanisms such as gut microbiota imbalance and oxidative stress [41]. Therefore, promoting healthy diets rich in whole grains, low in processed foods, and implementing stronger regulations on processed meat products could play a crucial role in reducing the burden of colorectal cancer. As the elderly population experiences a rising incidence of obesity, high BMI has become a key contributor to the growing burden of gallbladder and biliary tract cancers. Health promotion initiatives, including educational campaigns and community empowerment, are crucial for changing lifestyle and dietary habits to prevent obesity [42]. Elevated FPG and smoking are recognized as major risk factors for pancreatic cancer, but their interactive effects remain unclear. A large nationwide cohort study in Korea, involving over 9 million adults, found that current smokers with diabetes or prediabetes had a synergistically increased risk of pancreatic cancer (HR = 2.72) compared to never-smokers with normoglycemia [43]. In contrast, a 10-year prospective cohort study of 446,407 Korean men confirmed that smoking and elevated fasting glucose act as independent risk factors for pancreatic cancer [43].

From 1990 to 2021, the global burden of esophageal cancer, stomach cancer, gallbladder cancer, and biliary tract cancer exhibited a declining trend. This positive change resulted from the combined effects of global public health interventions, risk factor control, and improvements in medical care. In particular, the eradication of Helicobacter pylori infection and the implementation of early endoscopic screening played crucial roles in reducing the burden of stomach and esophageal cancers [27,44,45]. Conversely, the global burden of pancreatic cancer rose steadily, with both incidence and mortality increasing, largely driven by population aging [46]. Moreover, due to the lack of effective early screening methods and the disease’s high fatality rate, both direct medical costs and indirect economic losses associated with pancreatic cancer steadily increased [47]. It was suggested that preventive strategies should prioritize primary prevention, focus on modifiable risk factors, such as reducing smoking and controlling obesity and diabetes, to mitigate the burden of disease [48,49].

For colon and rectum cancer, screening plays a dual role in driving the paradox of rising incidence and falling mortality. On one hand, the widespread adoption of fecal occult blood testing (FOBT) and colonoscopy in Western countries has significantly enhanced the detection of early lesions [50,51]. On the other hand, screening directly reduces mortality. Colonoscopy allows for the removal of precancerous polyps lower the risk of post-procedure colorectal cancer by blocking the disease at precancerous or early stages [52]. Additionally, optimized 5-fluorouracil-based adjuvant chemotherapy further improves outcomes in advanced cases [53]. Regarding liver cancer, the decline in mortality from virus-related hepatocellular carcinoma was largely attributed to hepatitis B vaccination programs, antiviral therapies, and improvements in chronic liver disease management [54]. However, with the rising prevalence of metabolic-associated fatty liver disease, the overall incidence of liver cancer continued to increase [55]. It was also noteworthy that the incidence of benign and in situ intestinal neoplasms rose sharply, predominantly in high-SDI countries. This trend reflected expanded screening coverage and increased public health awareness, leading to earlier detection of asymptomatic lesions. For example, in the Netherlands’ nationwide colon and rectum cancer screening program, numerous inflammatory polyps and early-stage tumors were detected [56,57].

High-SDI countries have made significant strides in reducing mortality from digestive system neoplasms, driven by comprehensive cancer screening, early diagnosis, and well-developed treatment systems [18]. In contrast, middle and low-SDI countries face persistent challenges due to limited screening coverage, inadequate chronic disease management, and constrained healthcare resources, leading to higher rates of late-stage diagnoses and sustained disease burden [58,59]. To alleviate the global burden of digestive system neoplasms in Middle-Aged and Elderly Adults, targeted policy and practical measures should be prioritized. First, cost-effective, population-based screening programs, such as FOBT for colorectal cancer or endoscopic screening for gastric cancer, should be tailored to local contexts, with an emphasis on expanding coverage for high-risk middle-aged and elderly populations in resource-limited settings. Second, primary prevention must be strengthened through public health policies promoting physical activity, scaling up tobacco cessation programs, and regulating harmful products like sugar-sweetened beverages to address metabolic risk factors such as obesity and hyperglycemia [60]. Third, healthcare capacity in low and middle-SDI regions must be strengthened by investing in diagnostic and treatment infrastructure, training healthcare professionals, and utilizing telemedicine to ensure timely access to care for elderly populations [61]. Finally, collaboration between academia, healthcare providers, and industry should be fostered to accelerate advances in early detection and precision medicine, exemplified by initiatives like the U.S. “Cancer Moonshot” [62]. By integrating these strategies, global efforts can address the burden of digestive system neoplasms in middle-aged and elderly populations, while also tackling the underlying metabolic drivers, leading to more equitable health outcomes.

This study, based on data from the GBD Study 2021, systematically assesses the global burden of digestive system neoplasms among middle-aged and elderly populations. Nevertheless, several limitations warrant consideration. First, in certain low- and middle-income countries, the limited availability of high-quality epidemiological data may compromise model-based estimates due to data gaps and diagnostic biases. Second, the GBD applies standardized disease definitions, which may not fully capture histological diversity or borderline cases, potentially leading to an underestimation of the true disease burden. Furthermore, the attribution of risk factors relies on literature reviews and theoretical minimum risk exposure levels, which may not encompass all relevant exposures or adequately reflect long-term, population-specific variations. Third, this study does not account for subnational heterogeneity, thereby limiting the generalizability of the findings to regional policy planning Future research should prioritize the integration of real-world data and longitudinal cohort studies to better characterize disease heterogeneity and population-specific features, ultimately informing the development of more targeted and effective prevention strategies.

## 5. Conclusions

In conclusion, digestive system neoplasms remain a major global public health issue among middle-aged and elderly populations, with clear temporal trends and regional disparities observed between 1990 and 2021. Despite significant progress in early screening and treatment, the mortality rates for colorectal cancer, liver cancer, and pancreatic cancer have declined, while the incidence of these cancers, along with pancreatic cancer and benign tumors, continues to rise. This trend is primarily attributed to changes in lifestyle and the increasing prevalence of metabolic risk factors. In contrast, the burden of esophageal cancer, gastric cancer, and gallbladder/biliary tract cancer has decreased, reflecting the effectiveness of public health interventions. With the aging global population, the burden of digestive system neoplasms is likely to increase further. Therefore, it is essential to strengthen early screening, health education, and risk factor management, as well as promote advances in precision medicine, particularly in low- and middle-SDI regions. Enhancing public health infrastructure and healthcare access is critical to mitigating this growing health burden.

## Supporting information

S1 Fig EAPC of death rates for digestive system neoplasms in global and regional populations of middle-aged and elderly adults (1990–2021). (A) Esophageal cancer. (B) Stomach cancer. (C) Gallbladder and biliary tract cancer. (D) Colon and rectum cancer. (E) Liver cancer. (F) Pancreatic cancer.
(TIF)

S2 Fig EAPC of DALY rates for digestive system neoplasms in global and regional populations of middle-aged and elderly adults (1990–2021). (A) Esophageal cancer. (B) Stomach cancer. (C) Gallbladder and biliary tract cancer. (D) Colon and rectum cancer. (E) Liver cancer. (F) Pancreatic cancer.
(TIF)

S3 Fig Death rates of digestive system neoplasms in middle-aged and elderly adults per 100,000 population for both sexes in 204 countries, in 2021. (A) Esophageal cancer. (B) Stomach cancer. (C) Gallbladder and biliary tract cancer. (D) Colon and rectum cancer. (E) Liver cancer. (F) Pancreatic cancer.
(TIF)

S4 Fig DALY rates of digestive system neoplasms in middle-aged and elderly adults per 100,000 population for both sexes in 204 countries, in 2021. (A) Esophageal cancer. (B) Stomach cancer. (C) Gallbladder and biliary tract cancer. (D) Colon and rectum cancer. (E) Liver cancer. (F) Pancreatic cancer.
(TIF)

S5 Fig Global counts and death rates of digestive system neoplasms in middle-aged and elderly adults by age and sex, 2021. (A) Esophageal cancer. (B) Stomach cancer. (C) Gallbladder and biliary tract cancer. (D) Colon and rectum cancer. (E) Liver cancer. (F) Pancreatic cancer.
(TIF)

S6 Fig Global counts and DALY rates of digestive system neoplasms in middle-aged and elderly adults by age and sex, 2021. (A) Esophageal cancer. (B) Stomach cancer. (C) Gallbladder and biliary tract cancer. (D) Colon and rectum cancer. (E) Liver cancer. (F) Pancreatic cancer.
(TIF)

S7 Fig Temporal trends of age-standardized incidence rates for digestive system neoplasms in middle-aged and elderly adults (1990–2035). (A) Esophageal cancer. (B) Stomach cancer. (C) Colon and rectum cancer. (D) Liver cancer. (E) Gallbladder and biliary tract cancer. (F) Pancreatic cancer. (G) Benign and in situ intestinal neoplasms.
(TIF)

S8 Fig Temporal trends of age-standardized death rates for digestive system neoplasms in middle-aged and elderly adults (1990–2035). (A) Esophageal cancer. (B) Stomach cancer. (C) Gallbladder and biliary tract cancer. (D) Colon and rectum cancer. (E) Liver cancer. (F) Pancreatic cancer.
(TIF)

S1 Table The DALY counts and rates for digestive system neoplasms in global and regional populations of middle-aged and elderly adults in 2021, and the definition of DALY. (XLSX)

S2 Table ICD-10 and ICD-9 codes mapped to seven digestive system neoplasms for death and non-fatal causes. (XLSX)

S3 Table Definitions of risk factors associated with seven digestive system neoplasms and their TMREL. (XLSX)

S4 Table The incidence counts and rates for digestive system neoplasms in global and regional populations of middle-aged and elderly adults, in 2021. (XLSX)

S5 Table The mortality counts and rates for digestive system neoplasms in global and regional populations of middle-aged and elderly adults, in 2021. (XLSX)

S1 File The results of the global, regional, and national trends, as well as the age and sex patterns of benign and in situ intestinal neoplasms. (DOCX)
