# Supplementary figures and images for: Burden of digestive system neoplasms in middle-aged and elderly adults: Temporal trends and geographic disparities (1990–2021)
Source: PLoS One. 2025 Aug 21;20(8):e0330259. doi: 10.1371/journal.pone.0330259 (PMC12370137; doi:10.1371/journal.pone.0330259)

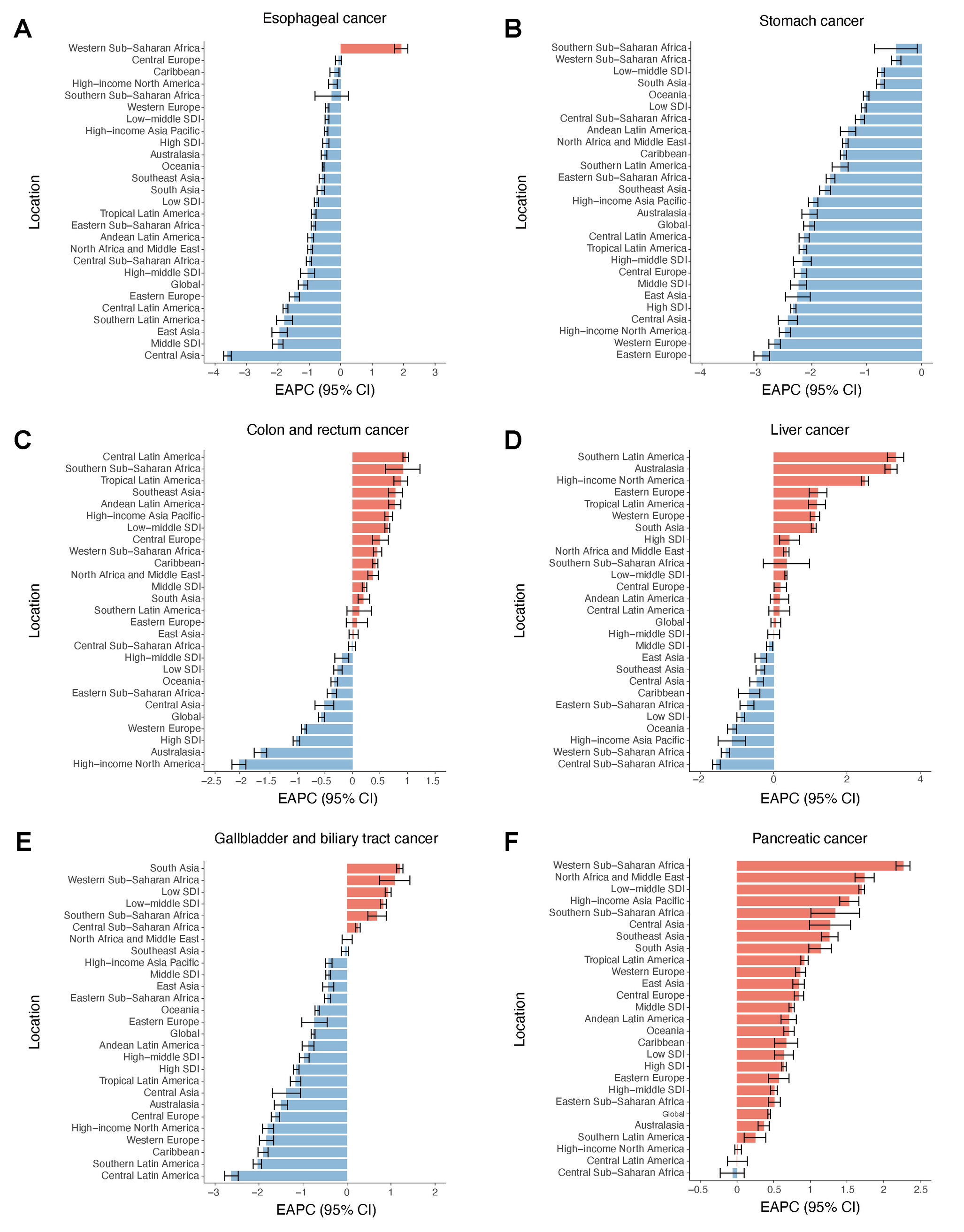

Supplement: S1 Fig — (A) Esophageal cancer. (B) Stomach cancer. (C) Gallbladder and biliary tract cancer. (D) Colon and rectum cancer. (E) Liver cancer. (F) Pancreatic cancer. (TIF) [file pone.0330259.s001.tif]

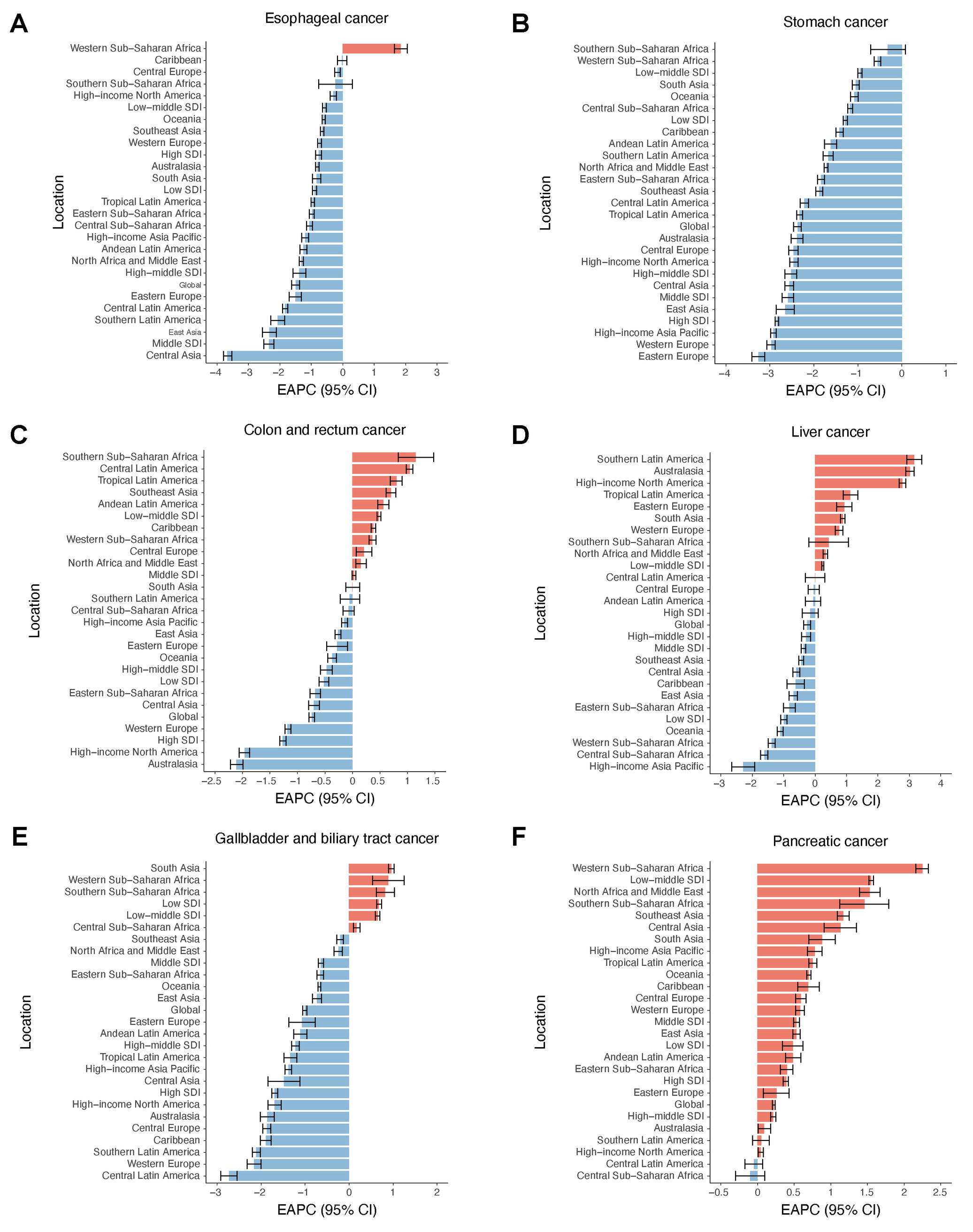

Supplement: S2 Fig — (A) Esophageal cancer. (B) Stomach cancer. (C) Gallbladder and biliary tract cancer. (D) Colon and rectum cancer. (E) Liver cancer. (F) Pancreatic cancer. (TIF) [file pone.0330259.s002.tif]

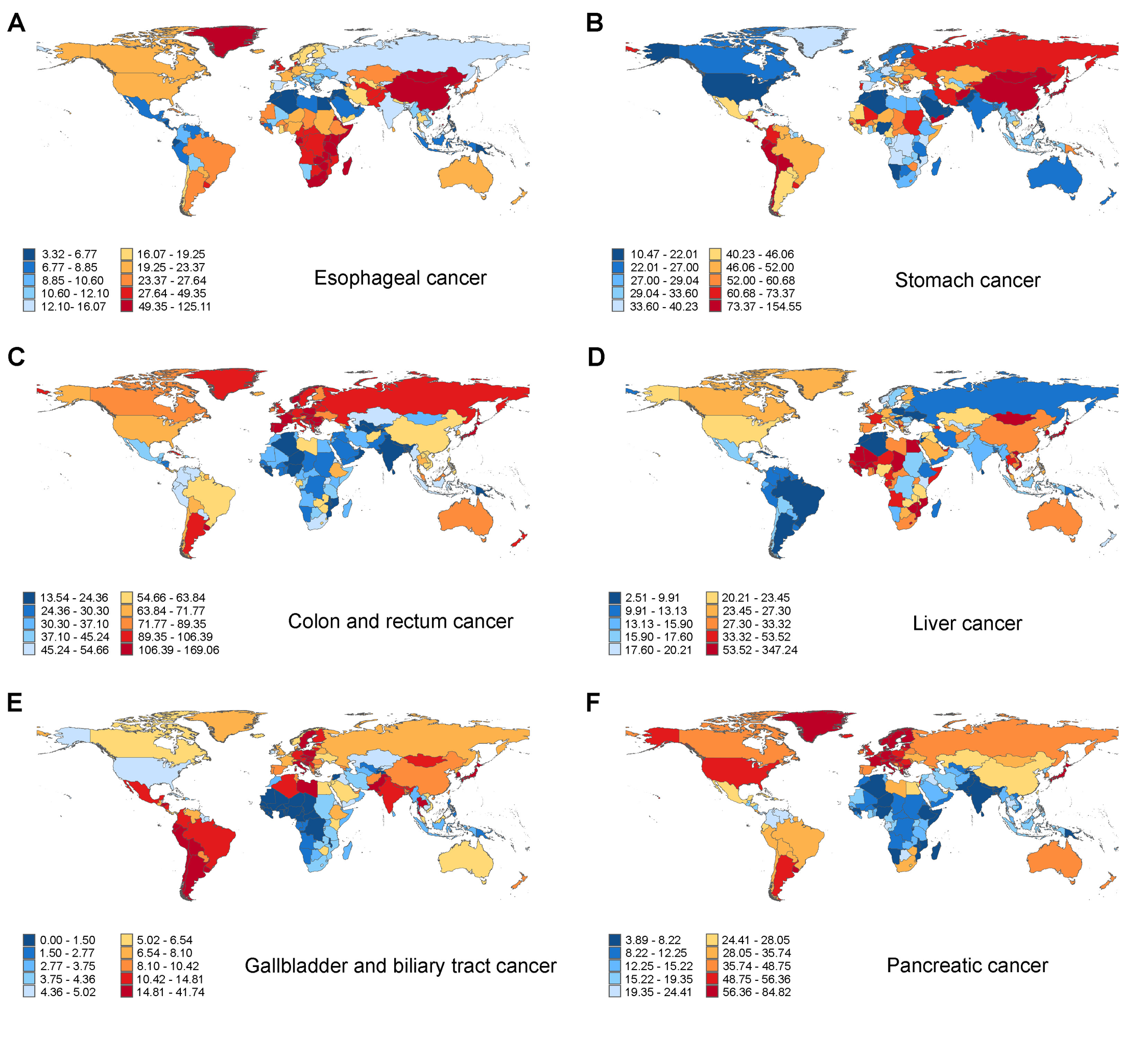

Supplement: S3 Fig — (A) Esophageal cancer. (B) Stomach cancer. (C) Gallbladder and biliary tract cancer. (D) Colon and rectum cancer. (E) Liver cancer. (F) Pancreatic cancer. (TIF) [file pone.0330259.s003.tif]

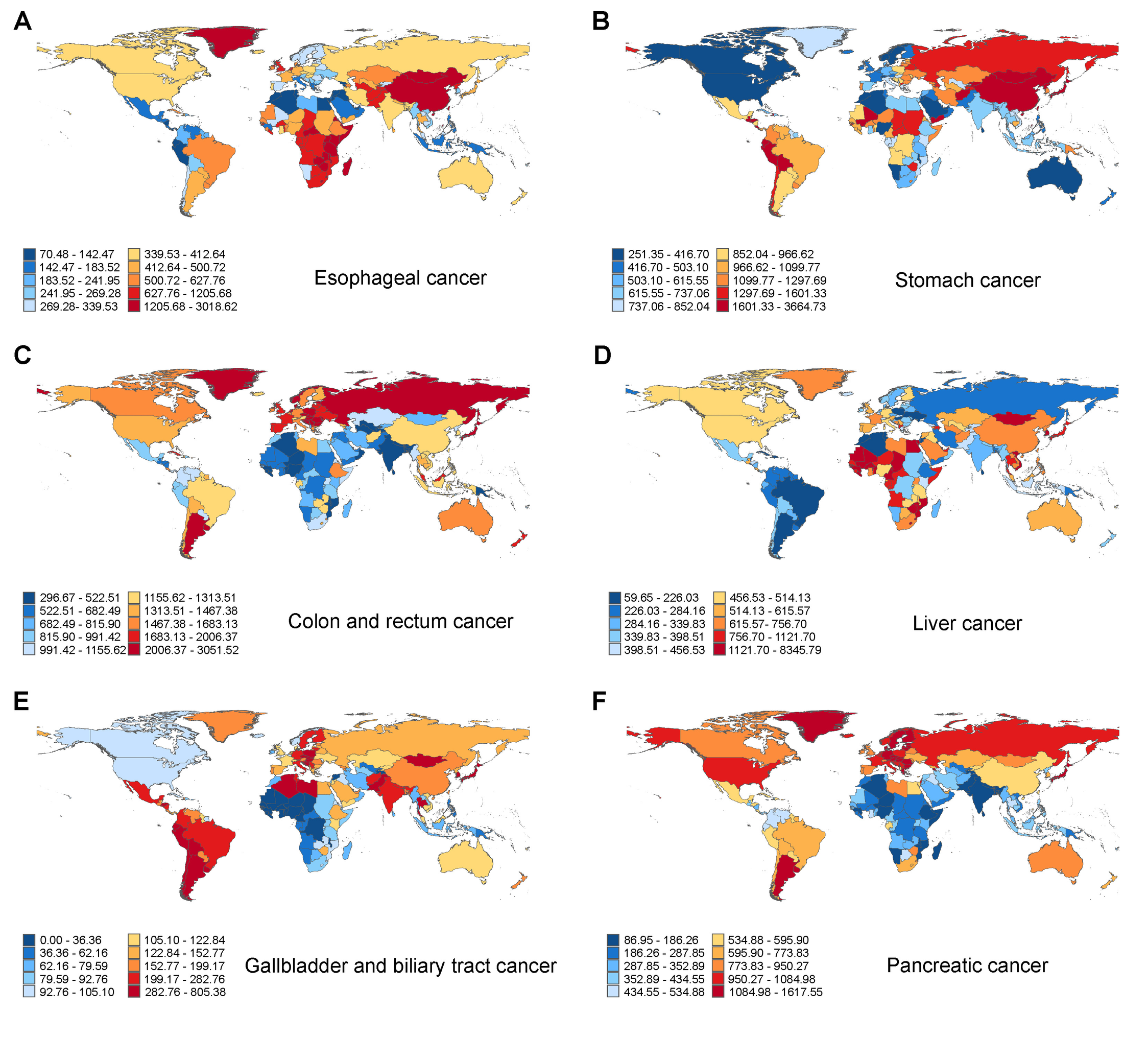

Supplement: S4 Fig — (A) Esophageal cancer. (B) Stomach cancer. (C) Gallbladder and biliary tract cancer. (D) Colon and rectum cancer. (E) Liver cancer. (F) Pancreatic cancer. (TIF) [file pone.0330259.s004.tif]

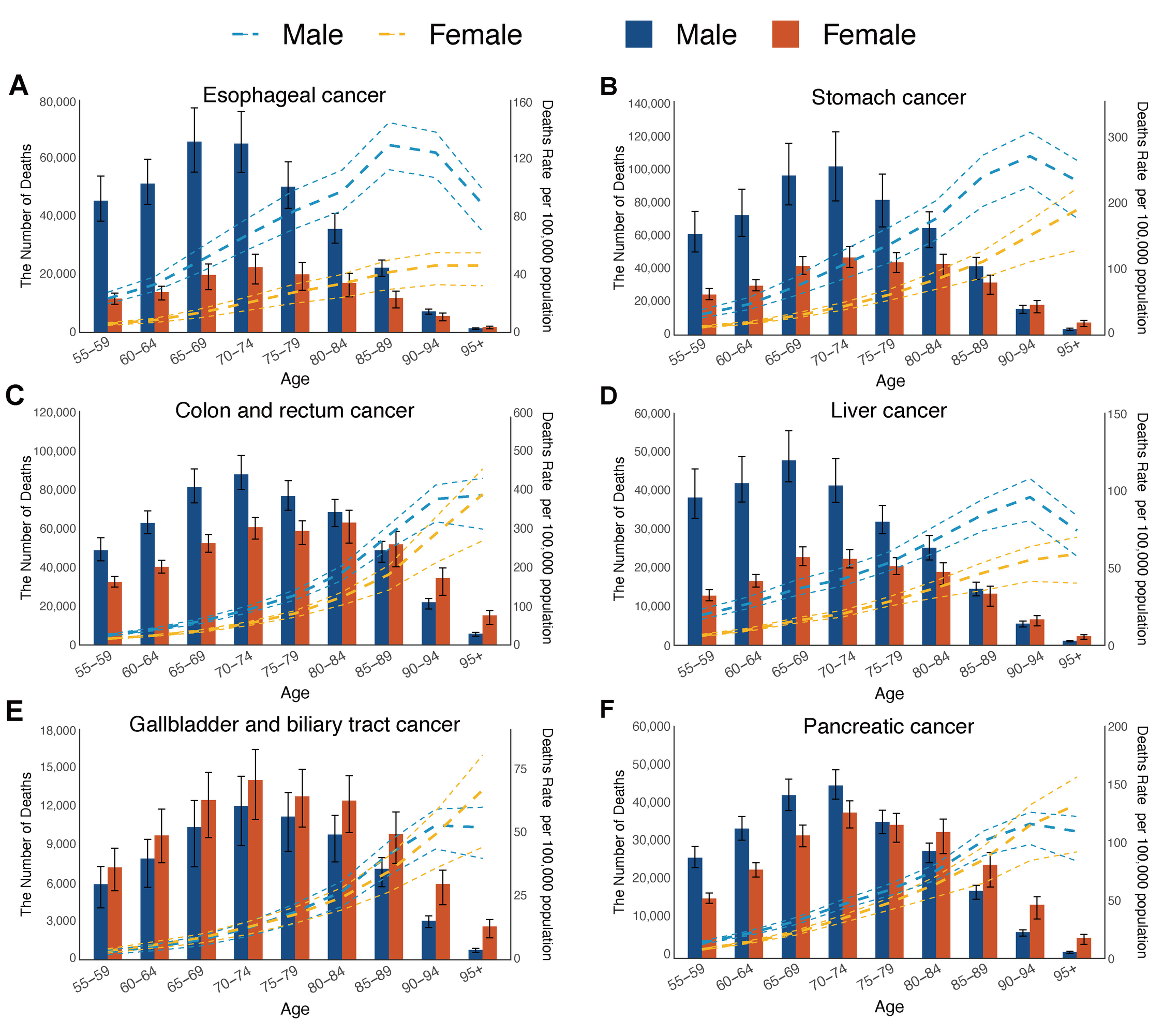

Supplement: S5 Fig — (A) Esophageal cancer. (B) Stomach cancer. (C) Gallbladder and biliary tract cancer. (D) Colon and rectum cancer. (E) Liver cancer. (F) Pancreatic cancer. (TIF) [file pone.0330259.s005.tif]

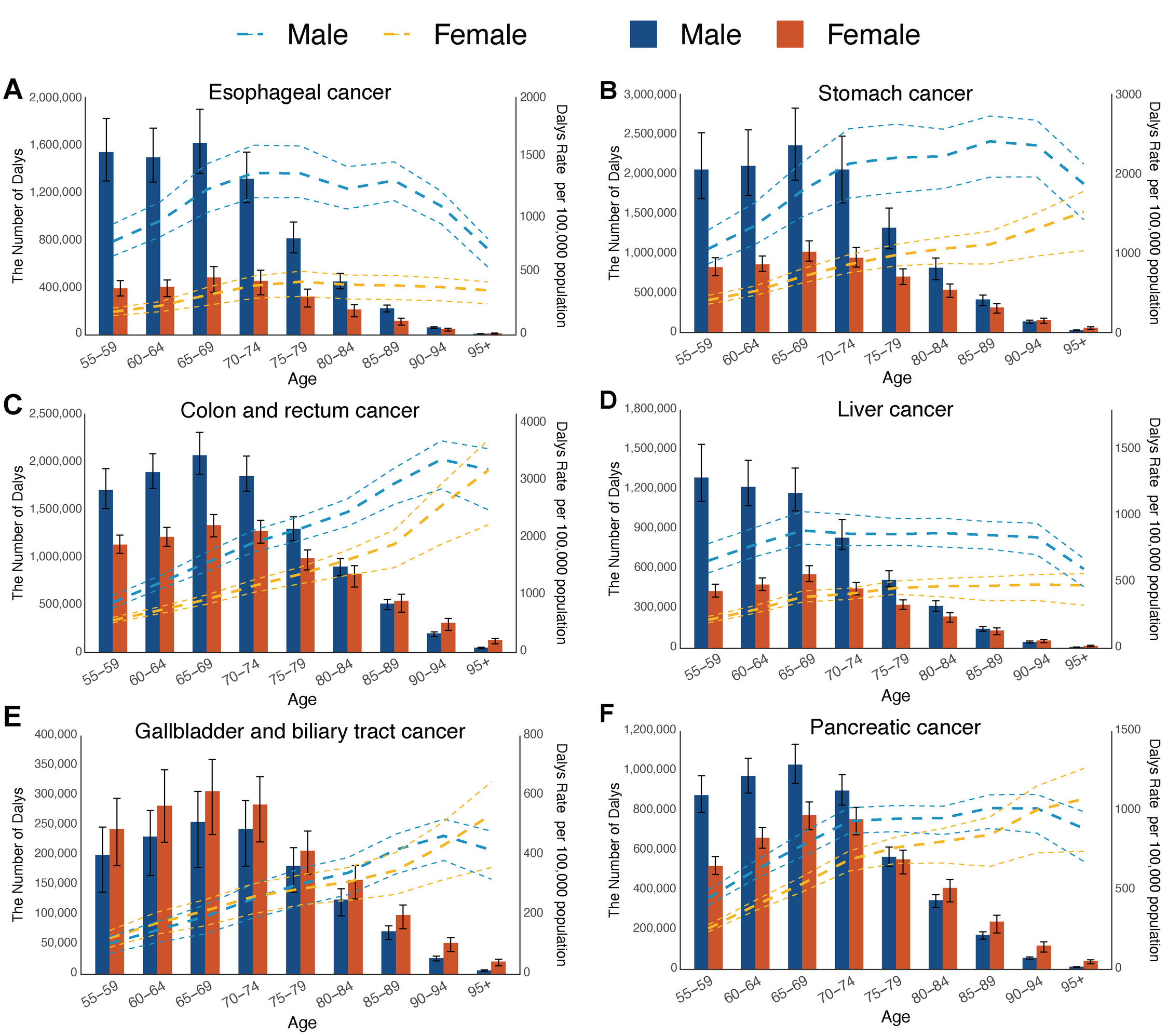

Supplement: S6 Fig — (A) Esophageal cancer. (B) Stomach cancer. (C) Gallbladder and biliary tract cancer. (D) Colon and rectum cancer. (E) Liver cancer. (F) Pancreatic cancer. (TIF) [file pone.0330259.s006.tif]

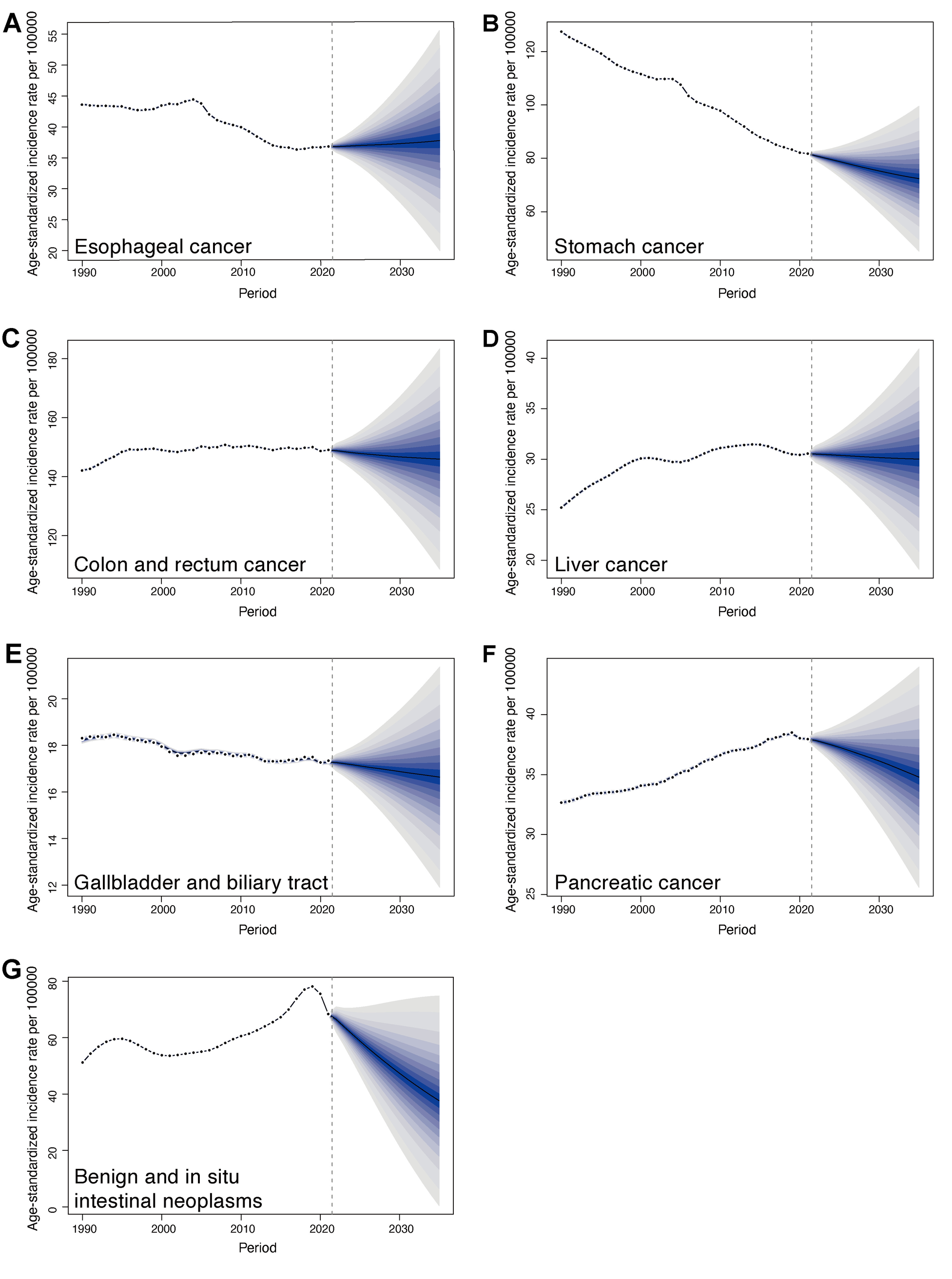

Supplement: S7 Fig — (A) Esophageal cancer. (B) Stomach cancer. (C) Colon and rectum cancer. (D) Liver cancer. (E) Gallbladder and biliary tract cancer. (F) Pancreatic cancer. (G) Benign and in situ intestinal neoplasms. (TIF) [file pone.0330259.s007.tif]

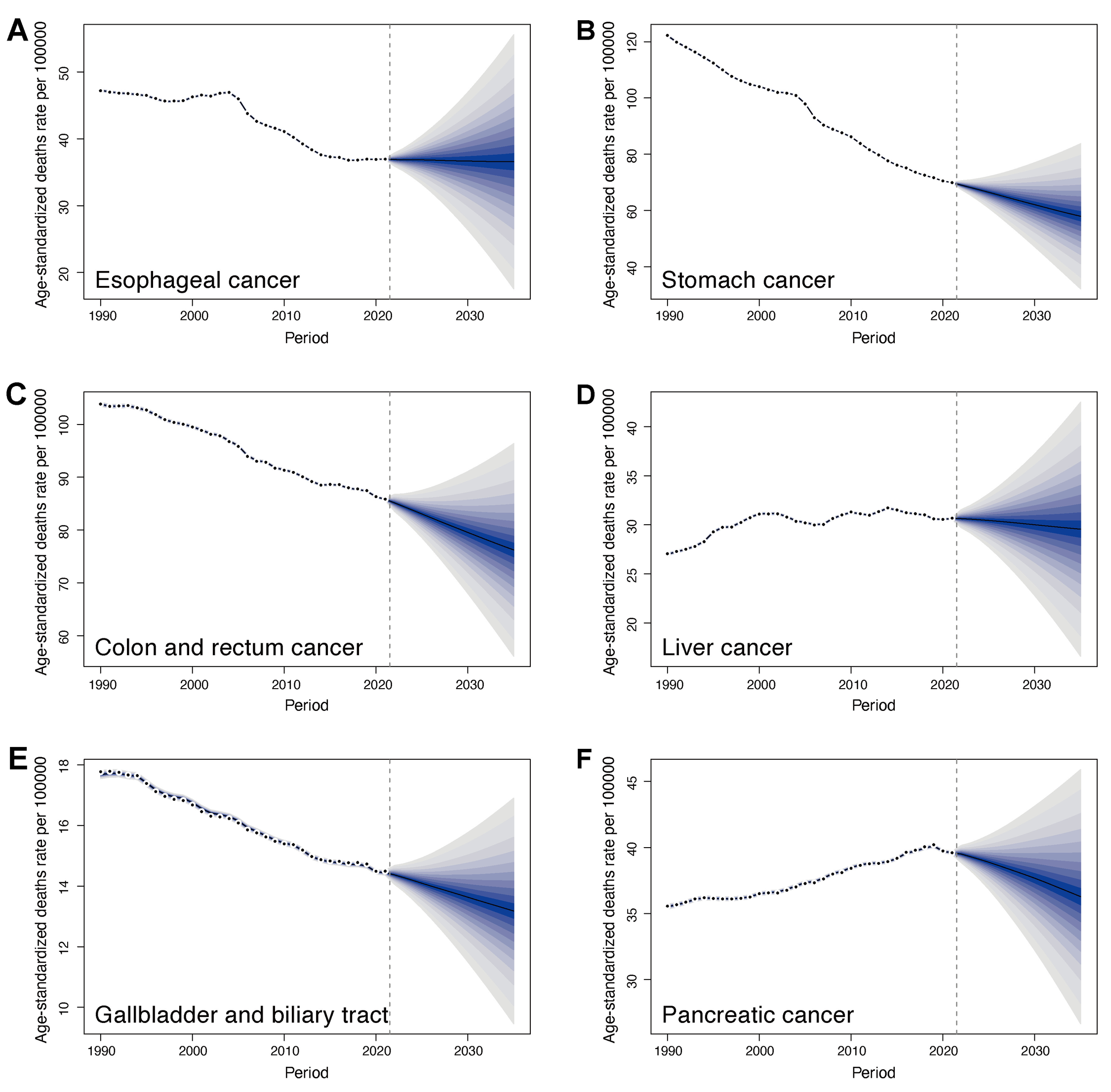

Supplement: S8 Fig — (A) Esophageal cancer. (B) Stomach cancer. (C) Gallbladder and biliary tract cancer. (D) Colon and rectum cancer. (E) Liver cancer. (F) Pancreatic cancer. (TIF) [file pone.0330259.s008.tif]
