# Supplementary material for: Burden of digestive system neoplasms in middle-aged and elderly adults: Temporal trends and geographic disparities (1990–2021)
Source: PLoS One. 2025 Aug 21;20(8):e0330259. doi: 10.1371/journal.pone.0330259 (PMC12370137; doi:10.1371/journal.pone.0330259)
Supplement: S1 File — (DOCX) [file pone.0330259.s014.docx]

**The results of the global, regional, and national trends, as well as the age and sex patterns of benign and in situ intestinal neoplasms.**

**Result**

**3.1 Benign and in situ intestinal neoplasms in Middle-Aged and Elderly Adults: Global Trends**

In 2021, the global incidence of benign and in situ intestinal neoplasms was estimated at 1,038,322 (95% UI 744,432 to 1,431,781), with an incidence rate of 69.87 per 100,000(95% UI 50.1 to 96.35). The incidence of these neoplasms demonstrated a upward trend (Table 1).

**3.2 Benign and in situ intestinal neoplasms in Middle-Aged and Elderly Adults: Regional Level**

Benign and in situ intestinal neoplasms had the highest incidence rate (543.36 per 100,000, 95% UI 387.02 to 748.19) in High-income North America in 2021 (Table S1). From 1990 to 2021, the incidence rate of benign tumors in this region was also observed the most rapid increase (Fig. 1).

**3.3 Benign and in situ intestinal neoplasms in Middle-Aged and Elderly Adults: National Trends**

In 2021, the incidence rate of benign and in situ intestinal neoplasms was highest in the United States of America at 572.45 per 100,000(95% UI 788.09 to 408.97), while the lowest incidence rate was in Eritrea at 5.33 per 100,000(95% UI 8.03 to 3.59) (Fig. 2).Notably, China led globally in the number of incidences, mortality, and DALYs for gastrointestinal neoplasms across all subtypes in 2021, except for the incidence number of benign and in situ intestinal neoplasms, which was highest in the United States (Fig. 2 and S3-S4).

**3.4 Benign and in situ intestinal neoplasms in Middle-Aged and Elderly Adults: Age and Sex Patterns**

As age increased, the trends in the incidence numbers and rates of benign and in situ intestinal neoplasms were similar for both genders. The number of cases was concentrated in the 65–69 age group, while the incidence rate peaked in the 70–75 age group (Fig. 3).
